# Exploring Novel Treatment Modalities for Type 1 Diabetes Mellitus: Potential and Prospects

**DOI:** 10.3390/healthcare12151485

**Published:** 2024-07-26

**Authors:** Rasha Aziz Attia Salama, Mohamed Anas Mohamed Faruk Patni, Shadha Nasser Mohammed Ba-Hutair, Nihal Amir Wadid, Mushirabanu Sharifmiyan Akikwala

**Affiliations:** 1 Department of Community Medicine, College of Medicine, Ras Al Khaimah Medical and Health Science University, Ras Al Khaimah 11172, United Arab Emirates; rasha.aziz@rakmhsu.ac.ae (R.A.A.S.); nihal.21901081@rakmhsu.ac.ae (N.A.W.); 2 Kasr El Aini Faculty of Medicine, Cairo University, Giza 12525, Egypt; 3 Department of Obstetrics and Gynecology, College of Medicine, Ras Al Khaimah Medical and Health Science University, Ras Al Khaimah 11172, United Arab Emirates; shadha@rakmhsu.ac.ae; 4 Shifa Al Jazeera Clinic, Ras Al Khaimah, United Arab Emirates; drmushira87@gmail.com

**Keywords:** type 1 diabetes mellitus, mesenchymal stem cells, gene therapy, pancreatic islet cell transplantation, teplizumab

## Abstract

Despite the effectiveness of insulin injections in managing hyperglycemia in type 1 diabetes mellitus (T1DM), they fall short in addressing autoimmunity and regenerating damaged islets. This review aims to explore the potential and prospects of emerging treatment modalities for T1DM, including mesenchymal stem cells (MSCs), MSC-derived exosomes, gene therapy, islet allotransplantation, pancreatic islet cell transplantation, and teplizumab. We review emerging treatment modalities for T1DM, highlighting several promising strategies with varied mechanisms and outcomes. Mesenchymal stem cells demonstrate potential in modulating the immune response and preserving or restoring beta-cell function, although variability in sources and administration routes necessitates further standardization. Similarly, MSC-derived exosomes show promise in promoting beta-cell regeneration and immune regulation, supported by early-stage studies showing improved glucose homeostasis in animal models, albeit with limited clinical data. Gene therapy, utilizing techniques like CRISPR-Cas9, offers targeted correction of genetic defects and immune modulation; however, challenges in precise delivery and ensuring long-term safety persist. Islet allotransplantation and pancreatic islet cell transplantation have achieved some success in restoring insulin independence, yet challenges such as donor scarcity and immunosuppression-related complications remain significant. Teplizumab, an anti-CD3 monoclonal antibody, has demonstrated potential in delaying T1DM onset by modulating immune responses and preserving beta-cell function, with clinical trials indicating prolonged insulin production capability. Despite significant progress, standardization, long-term efficacy, and safety continue to pose challenges across these modalities. **Conclusion:** While these therapies demonstrate significant potential, challenges persist. Future research should prioritize optimizing these treatments and validating them through extensive clinical trials to enhance T1DM management and improve patient outcomes.

## 1. Introduction

Diabetes mellitus (DM) stands as a chronic metabolic disorder that is increasingly prevalent worldwide. It is marked by either a partial or complete deficiency in insulin secretion, leading to persistently elevated blood glucose levels. This condition often manifests with an array of complications, including macrovascular issues like coronary heart disease and hypertension, as well as microvascular complications such as diabetic foot, diabetic encephalopathy, and diabetic kidney injury. These complications significantly diminish patients’ quality of life and decrease their chances of survival. The global impact of diabetes is substantial, imposing a significant economic and systemic burden on healthcare systems. It is estimated that diabetes affects approximately 422 million people worldwide, the majority living in low- and middle-income countries, and 1.5 million deaths are directly attributed to diabetes each year [1]. Type 1 diabetes, affecting five to ten percent of diabetes cases [2], has historically been dubbed “juvenile diabetes” or insulin-dependent diabetes due to its early onset. It is a chronic condition in which the pancreas produces little or no insulin by itself. For people living with diabetes, access to affordable treatment, including insulin, is critical to their survival. Specifically, both the number of cases and the prevalence of diabetes have been steadily increasing over the past few decades. Management requires life-long insulin replacement with multiple daily insulin injections, insulin pump therapy, or the use of an automated insulin delivery system. In addition to insulin therapy, glucose monitoring with a continuous glucose monitor (CGM) and a blood glucose monitor if a CGM is unavailable is recommended. Furthermore, self-management education and support should incorporate training in glucose monitoring, insulin administration, ketone testing when necessary, nutritional guidance on carbohydrate counting, physical activity, and strategies for preventing and treating hypoglycemia [3].

However, despite the effectiveness of insulin injections in managing hyperglycemia, they fall short in addressing autoimmunity and regenerating damaged islets, which are crucial in type 1 diabetes management. T1DM is a T-cell-mediated, organ-specific autoimmune disorder which is characterized by beta-cell destruction and decreased insulin production [4]. The pathogenesis of T1D involves the destruction of insulin-releasing pancreatic beta cells, with cellular invasion by both CD4+ and CD8+ T cells, resulting in the reduction in beta-cell mass [5]. Environmental exposures, in conjunction with genetic predispositions, play pivotal roles in transitioning from islet autoimmunity to full-fledged T1DM. Although researchers have identified more than 50 genetic loci associated with the disease, HLA polymorphisms contribute substantially, accounting for 40–50% of T1D cases [6,7].

The escalating prevalence of type 1 diabetes on a global scale has spurred significant advancements in its treatment over the past two decades. This trend has been particularly driven by two key factors: a focus on improving the quality of life for individuals with T1D and the growing recognition of the potential of regenerative medicine approaches to transform diabetes treatment.

**Figure 1:**
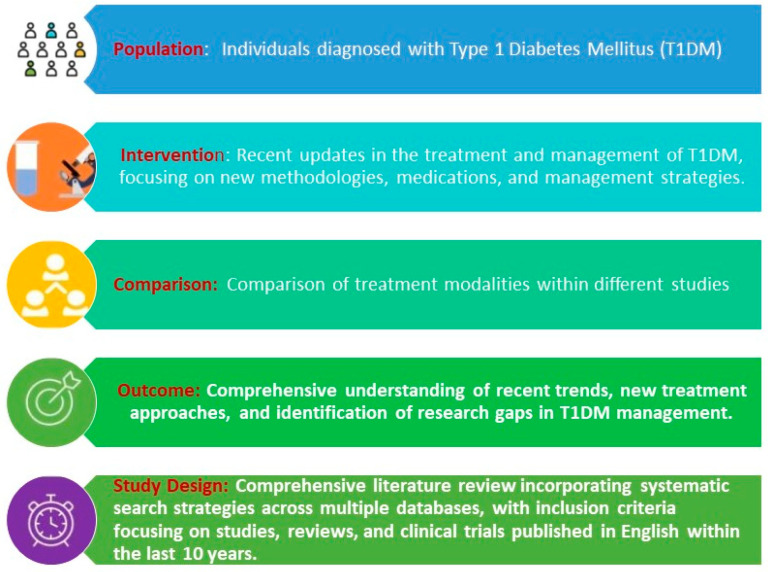
PICOS framework adapted for reviewing the literature.

Among these, progress has been achieved in areas such as mesenchymal stem cells (MSCs), gene therapy, pancreatic islet cell transplantation, and a new drug teplizumab, leveraging immune modulation strategies, beta-cell regeneration, and enhancing engraftment success rates. This review aimed to explore the emerging landscape of innovative treatment modalities for type 1 diabetes mellitus (T1DM), shedding light on both their potential and prospects. By offering actionable recommendations to stakeholders in healthcare, research, and policy domains, our goal is to catalyze progress in the field and improve outcomes for individuals living with T1DM. Following is the PICOS framework adapted for systematically reviewing the literature as shown in Figure 1.

## 2. Materials and Methods

A comprehensive review was conducted based on a search of the relevant literature. Unlike systematic reviews, narrative reviews are not intended to be comprehensive. To ensure a comprehensive literature search, four electronic databases were selected based on their relevance and coverage of medical and clinical research, PubMed, ProQuest, Google Scholar, and Scopus. A systematic search strategy was developed to identify relevant articles on recent updates in the treatment of type 1 diabetes mellitus. The search was conducted using specific keywords and Boolean operators to maximize the retrieval of pertinent articles. The keywords were chosen to capture a wide range of relevant studies. These included terms related to novelty, treatment approaches, and the condition itself: Novelty: “Novel”, “Recent”, “Updates”; Treatment: “Treatment”, “Management”, “Medications”; and Condition: “Type 1 Diabetes Mellitus”, “Diabetes Mellitus”, “Diabetes Mellitus Type 1” The identified keywords were combined using Boolean operators to create a comprehensive search string: (“Novel” OR “Recent” OR “Updates”) AND (“Treatment” OR “Management” OR “Medications”) AND (“Type 1 Diabetes Mellitus” OR “Diabetes Mellitus” OR “Diabetes Mellitus Type 1”). 

To ensure the relevance and quality of the selected articles, the following criteria were applied: Articles had to be published in English, with a preference for those published within the last 10 years to ensure the inclusion of recent updates. The focus was on studies, reviews, and clinical trials related to the treatment and management of type 1 diabetes mellitus. Articles not related to type 1 diabetes mellitus, studies not focused on treatment or management, duplicate articles across different databases, and non-English articles were excluded.

The results obtained from the database searches were managed systematically to ensure organization and ease of access for further analysis. Duplicate articles retrieved from multiple databases were identified and removed. A citation management tool was used to organize the articles. Zotero 6 was chosen for its ability to handle large datasets and facilitate easy retrieval and the referencing of articles. The organized articles were then reviewed for their content and relevance. The abstracts of the retrieved articles were reviewed to assess their relevance to the topic. Full-text articles were obtained for those that met the inclusion criteria based on the abstract review. The selected full-text articles were critically appraised for their methodology, findings, and contribution to the current understanding of type 1 diabetes mellitus treatment. Preference was given to high-quality studies, such as randomized controlled trials, systematic reviews, and meta-analyses. The findings from the selected articles were synthesized to provide a comprehensive overview of recent updates in the treatment of type 1 diabetes mellitus. This synthesis aimed to identify trends, new treatment approaches, and gaps in the current research.

**Figure 2:**
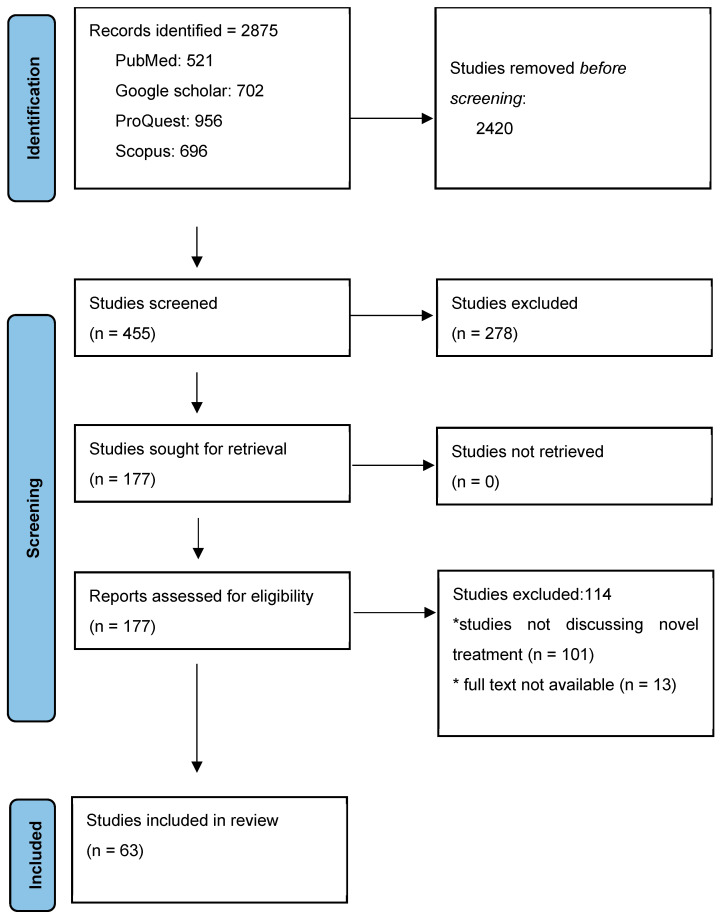
PRISMA flow diagram. * represents why studies were excluded and its already mentioned in flow chart.

The study made it possible to analyze 63 scientific articles published preferably in last 10 years related to the novel treatment of diabetes mellitus. The PRISMA flow diagram of each stage of the article selection process is described in the flowchart shown in Figure 2.

### 2.1. Standard Treatment by Insulin

The current standard treatment for type 1 diabetes mellitus (T1DM) is lifelong insulin replacement therapy. Insulin therapy is aimed to mimic the normal physiological secretion of insulin in response to meals and maintain blood glucose levels within a normal range.

The different types of insulin that can be used for T1DM include rapid-acting, short-acting, intermediate-acting, and long-acting insulin. The insulin type and regimen chosen depend on several factors, including the patient’s blood glucose control, lifestyle, and individual needs. Another essential constituent of T1DM is the self-monitoring of blood glucose (SMBG). This involves testing blood glucose levels regularly, usually multiple times per day, to help adjust insulin doses and monitor blood glucose control.

Additionally, managing type 1 diabetes mellitus (T1DM) involves implementing lifestyle modifications, such as adopting healthy eating habits and maintaining regular physical activity. It is recommended that individuals with T1DM consume balanced meals with an appropriate carbohydrate intake, timed alongside insulin doses, and incorporate physical activity into their routine. Moreover, education about diabetes and providing support are crucial aspects of T1DM management. Comprehensive education and training on the self-management of T1DM, including insulin administration, self-monitoring of blood glucose (SMBG), adopting healthy dietary patterns, engaging in regular physical activity, and effectively managing hypo- and hyperglycaemia, should be provided to patients and their families [3]. This holistic approach empowers individuals with T1DM to effectively manage their condition and improve their overall quality of life.

Despite significant advancements in insulin, insulin delivery, and glucose monitoring technology, many people with type 1 diabetes still do not achieve their glycemic goals. The substantial burden of type 1 diabetes on patients and their families must be acknowledged. Major challenges include calculating and timing prandial insulin doses, often from foods with unknown carbohydrate content, adjusting food and insulin during exercise, and managing the cost of therapy. The extensive impact of type 1 diabetes can lead to long-term complications, decreased life expectancy, reduced quality of life, and significant financial strain. Additionally, the psychological stress of acute management and the potential for chronic complications further contribute to the burden. This warrants education programs and regular monitoring for “diabetes burnout”, which are essential for everyone with type 1 diabetes.

Future perspectives in insulin replacement therapy including the use of artificial pancreas, immune modulation, stem cell mobilization, β cell encapsulation, and incretins could improve the graft survival rate and revascularization in transplanted islets. In certain situations, additional medications, such as GLP-1 receptor agonists or SGLT2 inhibitors, are added to insulin therapy to improve blood glucose control and reduce the risk of complications. However, insulin therapy remains the mainstay of T1DM management [2,8].

### 2.2. Mesenchymal Stem Cells

The future of diabetes treatment is centered around promoting beta-cell regeneration, achievable through self-replication or differentiation from progenitor cells via stem cell therapy. This approach not only tackles autoimmunity but also enhances endogenous insulin production. Multipotent Stem Cells (MSCs) emerge as a promising tool for these strategies, found primarily in the bone marrow but also in various other tissues [9]. MSCs possess the capability of in vitro differentiation into insulin-secreting cells, alongside immunomodulatory effects and the secretion of growth factors and cytokines [10]. They have demonstrated the ability to differentiate into multiple cell types, including islet cells, depending on specific signaling pathways. Notably, MSCs exhibit hypo-immunogenicity and can be home to injured tissue, making them efficient carriers for therapeutic proteins [11]. Traditionally, combating type 1 diabetes (T1D) involves depleting antibodies against T cells, but this method lacks specificity and may lead to complications. MSCs offer an alternative approach by secreting soluble factors that modulate immune responses, providing greater selectivity in targeting hyper-reactive T cells [12]. Clinical trials have shown reduced levels of islet cell antibodies after MSC treatment, suggesting potential immunomodulatory effects. MSCs also induce the production of regulatory T cells, restoring immune balance and protecting beta cells against damage [13,14].

While the direct transformation of MSCs into beta cells remains unclear, systemic treatment with MSCs has been shown to increase beta cell mass and reverse hyperglycemia in streptozotocin-induced diabetic rats [15]. These findings suggest the potential of MSCs in promoting beta cell regeneration and improving glucose metabolism, offering promise for T1D treatment.

Several successful attempts have been made to generate insulin-producing cells in vitro through the trans-differentiation of MSCs. The first report in 2004 showcased the in vitro trans-differentiation of rat MSCs into functional insulin-producing islet cells, effectively controlling blood glucose levels in diabetic rats [16]. The transcription factor Pdx-1 played a pivotal role in facilitating the efficient trans-differentiation of MSCs into beta cells [9,17,18].

Other studies have elucidated the roles of various transcription factors such as paired box gene 4 (PAX4), neurogenin 3 (Ngn3), forkhead box protein A2 (FOXA2), hepatocyte nuclear factor 6 (HNF6), glucagon-like peptide-1 (GLP-1), and epidermal growth factor (EGF) in promoting beta cell regeneration [9,19,20,21,22]. Additionally, MSCs have been proposed as a potential source of “artificial” human islets in vitro due to their ability to differentiate into glucagon and somatostatin-expressing cells [9,23]. Subsequent studies have further demonstrated the formation of islet-like clusters in vitro from MSCs with appropriate stimulation [7,22,23]. These findings underscore the potential of MSCs in generating insulin-producing cells and advancing the field of diabetes treatment. 

Recent advancements in tissue engineering have highlighted the potential necessity of a biocompatible scaffold for the in vitro generation of artificial islets with functional vasculature from stem cells [24]. However, it is crucial to approach these reports with caution due to the challenges encountered, including the failure to generate functional islets as a whole. This failure can disrupt the balance between insulin and glucagon, potentially leading to complications. 

Despite the potential of MSCs in the treatment of T1DM, several challenges and debates surround its use, particularly concerning its effectiveness and long-term impact. One significant challenge is the potential for immune rejection; while MSCs are generally considered immune-privileged, there is still a risk of immune response in some patients. Variability between donor MSCs can lead to inconsistent therapeutic outcomes due to differences in donor age, health status, and cell processing techniques [25]. MSCs often exhibit limited engraftment in the host tissue, which reduces their potential efficacy as they may not integrate well into pancreatic tissue or may not survive long enough to exert a lasting therapeutic effect [26]. Even when MSCs initially engraft and function, their effects may be transient, with some studies suggesting that therapeutic effects diminish over time. There are also concerns about the potential for MSCs to form tumors, as their manipulation and proliferation could potentially lead to tumorigenesis [26]. The long-term safety profile of MSC therapy is not fully established, raising concerns about adverse effects such as ectopic tissue formation and unintended differentiation [27].

The literature presents conflicting data on the effectiveness of MSC therapy for T1DM. Some studies report significant improvements in insulin production and glycemic control, while others find minimal or no benefit, with differences in study design, MSC source, dosage, and administration route contributing to the variability in reported outcomes [28]. The precise mechanisms by which MSCs exert their therapeutic effects are not fully understood, though it is believed that MSCs modulate the immune response and promote tissue repair. The heterogeneity in MSC research, including differences in cell isolation, culture, and administration protocols, poses a significant obstacle to obtaining consistent and comparable results [29]. Regulatory hurdles also impede progress, as differences in regulatory requirements across countries can delay the development and clinical application of these therapies [26]. Ethical concerns regarding the source of MSCs, particularly those derived from embryonic tissues, can limit their use and acceptance [30]. Additionally, the high cost of MSC therapy and logistical challenges in cell production and delivery limits its accessibility to patients, necessitating the development of cost-effective and scalable production methods for broader applications [28]. Addressing these challenges through standardized protocols and comprehensive research is essential for realizing the potential of MSC therapy in T1DM care. Further research and collaboration among scientists, clinicians, and policymakers are needed to enhance therapeutic outcomes for individuals living with T1DM.

Moreover, Mesenchymal Stem Cell Exosomes (MSC-Exos) emerge as another promising approach for managing diabetes mellitus. These extracellular vesicles, possessing the same bi-lipid cell membrane as MSCs, have demonstrated effectiveness in various studies focused on tissue repair, including spinal cord, kidney, liver, cardiovascular, and skin injuries [31,32,33]. MSC-Exos have been shown to promote the regeneration of pancreatic beta cells, enhance insulin secretion, and improve glucose metabolism, which are critical aspects in managing T1DM [34]. They exert immunomodulatory effects by promoting regulatory T cells (Tregs) and suppressing pro-inflammatory cytokines, potentially attenuating the autoimmune destruction of pancreatic beta cells [35]. Additionally, MSC-Exos possesses anti-inflammatory and anti-apoptotic properties that contribute to preserving functional beta-cell mass, essential for maintaining insulin production and glucose homeostasis [36]. Their ability to deliver bioactive molecules selectively to pancreatic beta cells further enhances their therapeutic efficacy while minimizing off-target effects. The potential of MSC-Exos to address multiple facets of T1DM pathophysiology makes them a promising candidate for future therapeutic interventions, pending further research and clinical validation. However, significant challenges impact their potential as therapeutic agents. Key challenges include the need for standardized isolation and characterization methods, which affect the reproducibility and comparability of results in clinical applications [37]. There is ongoing debate regarding the precise mechanisms of action of MSC-Exos, including whether their therapeutic effects primarily result from cargo molecule delivery, modulation of cellular signaling pathways, or other interactions with recipient cells [38]. Safety concerns also persist, despite their generally low immunogenicity, with uncertainties surrounding potential immune reactions and long-term effects in clinical settings [39]. Additionally, the biological stability of MSC-Exos during storage and their sensitivity to freeze–thaw cycles pose logistical challenges that must be addressed to ensure their efficacy in therapeutic applications [40]. Economic factors, such as the scalability of production and cost-effectiveness, further limit the widespread accessibility of MSC-Exos therapies, emphasizing the need for advancements in production techniques and regulatory frameworks [40]. 

### 2.3. Gene Therapy

Gene therapy in T1DM holds significant promise, leveraging advanced techniques like CRISPR-Cas9 to offer the targeted correction of genetic defects and immune modulation. This therapeutic approach involves transferring engineered genetic material into human cells using viral or non-viral methods. In T1DM, various delivery vehicles are utilized, including viral vectors such as adenovirus and lentivirus, and non-viral vectors like plasmid DNA, liposomes, and polymeric nanoparticles. Each vehicle presents unique benefits and limitations, such as immunogenicity, size constraints, and gene transfer efficiency [41]. Given knowledge of the desired target whose function needs to be suppressed, the suppression of gene expression, along with gene replacement or augmentation, is beneficial for achieving the endpoint of gene therapy. Nucleic acids as recombinant plasmid DNA and replication-defective viruses have been the leading vectors in gene therapy trials [42]. Recent techniques involve the combination of small interfering RNA (siRNA) and peptide nucleic acids (PNAs) with nucleic acids [43]. In addition to these mainstays, protein duction domains, naked oligonucleotides, and liposome formulations have been utilized in gene therapy. However, viral vectors have been extensively used in gene therapy studies for type 1diabetes mellitus to engineer islets, beta-cell surrogates, immune cells, or specific anatomical sites [44].

The autoimmune response targeting beta cells in T1D suggests that certain factors from these cells could be harnessed to regulate the immune system. Beta-cell-restricted proteins have been identified as potential autoantigen triggers for T cells, indicating a possible avenue for intervention [43]. Additionally, introducing genetically modified or non-modified immune cells has shown promise in halting the progression of diabetes, highlighting the potential for immunomodulation in T1D therapy [45]. Clinical studies have demonstrated the effectiveness of various approaches, including the use of TGF-alpha, soluble IFN-gamma receptor, viral vectors encoding IL-4 and IL-10, and regulatory T-cells CD4+ CD25+, NK-T, and CD8+ CD282, in reducing the progression of T1D by suppressing auto-reactive T cells and amplifying regulatory T cells [44,46,47,48,49,50,51]. Transplantation of intact islet Langerhans cells, once considered impractical due to the need for multiple donors and lack of long-term control over blood sugar levels, has been revisited [52]. By locally expressing immunoregulatory genes and trophic factors such as VEGF and NGF, islet transplants can create a conducive environment for engraftment, while the expression of free-radical scavenging proteins and anti-inflammatory agents by islet cells can enhance graft survival [44]. Moreover, surrogate beta cells offer a promising approach by transferring glucoregulatory genes into tissues less susceptible to autoimmune attacks, such as hepatocytes, skeletal muscle cells, intestinal K cells, and hypothalamic-pituitary cells [44,53]. Transcription factors like PDX-1 and adenoviral vectors encoding betacellulin or PAX-4 have been shown to generate functional beta cells, leading to diabetes reversal [53,54]. Gene therapy can also modify non-beta cells like mesenchymal stem cells and hematopoietic stem cells into beta-cell lineage, offering potential surrogates [55,56]. 

While gene therapy offers a promising approach for treating T1DM by potentially restoring insulin production through genetic modification, its application is fraught with significant challenges and debates. The effectiveness and long-term impact of gene therapy for T1DM are uncertain, with conflicting data reported in the literature. Some studies show significant improvements in insulin independence and glycemic control, while others report only transient benefits or no substantial impact, highlighting the variability and need for further optimization of gene delivery methods, vector selection, and targeting strategies [57].

Technical challenges in gene therapy include the efficient and targeted delivery of therapeutic genes to pancreatic beta cells, long-term expression of these genes, and avoidance of off-target effects. Viral vectors, commonly used for gene delivery, can trigger immune responses and pose safety risks such as insertional mutagenesis, potentially leading to oncogenesis [58]. Non-viral vectors, while safer, often suffer from low transfection efficiency and transient gene expression, limiting their therapeutic potential.

Ethical and social concerns are prominent in the application of gene therapy for T1DM. Privacy issues arise from the collection and storage of genetic information, which could be misused or disclosed without consent. Ensuring informed consent is critical, as patients must fully understand the risks, benefits, and uncertainties associated with gene therapy. Additionally, fair access to advanced treatments like gene therapy poses a significant challenge, as the high costs associated with these treatments could exacerbate health disparities, making them accessible only to those who can afford them and leaving underserved populations without these potentially life-changing interventions. Regulatory and standardization issues further complicate the clinical translation of gene therapy. Regulatory agencies require extensive evidence of safety and efficacy, involving rigorous preclinical testing followed by multiple phases of clinical trials. This process is time-consuming and expensive, and the lack of standardized protocols for the isolation and characterization of gene therapy products affects the reproducibility and comparability of results [57]. The long-term monitoring of patients is essential to ensure consistent and safe outcomes, but these requirements can slow down the development and accessibility of gene therapy.

Debates and conflicting data in the literature on gene therapy for T1DM reflect ongoing uncertainties and challenges. Differences in study design, gene delivery methods, and patient populations contribute to varying results. Some researchers advocate for the use of autologous cells to minimize immune rejection, while others explore allogeneic cells engineered to evade the immune system. These differing approaches underscore the need for consensus on the most effective and safe strategies for gene therapy in T1DM [59].

### 2.4. Pancreatic Islet Cell Transplantation

Islet cell transplantation involves extracting pancreatic islet cells from deceased donors and implanting them into the liver of the recipient. Islet allotransplantation is similar to islet cell transplantation but typically involves multiple donors to achieve an adequate islet mass. Over the past thirty years, pancreatic islet isolation and transplantation techniques have turned from a rare, experimental procedure to a routine clinical procedure with predictable efficacy for selected patients with type 1 diabetes mellitus [60]. The treatment is offered only for selected patients with unstable T1DM and hypoglycemia unawareness, severe hypoglycemic episodes, and glycemic lability who cannot be stabilized successfully with intensive insulin, pumps, and/or continuous glucose monitoring therapies [61]. This minimally invasive procedure can now routinely result in long-term glycemic control with near normalization of HbA1c in the absence of severe hypoglycemic episodes [62].

Islet cell transplantation involves extracting pancreatic islet cells from deceased donors and implanting them into the liver of the recipient. Islet allotransplantation is similar to islet cell transplantation but typically involves multiple donors to achieve an adequate islet mass. The process entails careful isolation of islet cells from the pancreas, using enzyme mixtures to separate islets from exocrine tissues and subsequent implantation into the liver via a cannula inserted into the pancreatic duct [63].

Despite the progress made in islet transplantation, significant challenges persist. The microenvironment of transplanted sites, encompassing factors like vascularization, extracellular matrix composition, and tissue-resident immune cells, remains poorly understood, hampering efforts to identify optimal transplantation locations. Current research endeavors focus on developing tailored hydrogel-based materials and microdevices to create transplantation spaces, shield grafts from the immune system, and promote angiogenesis. However, achieving normal glycemia with cadaveric human islets or those derived from stem cells remains elusive, and the long-term survival of transplanted islets varies across transplantation sites, influenced by factors like aging and other health conditions. Moreover, uncertainties surround the effectiveness and safety of biomaterials used to encapsulate artificial islets created from stem cells, with concerns about post-implantation trans-differentiation, teratoma formation, or graft migration requiring careful consideration. To enhance the long-term efficacy and safety of islet cell treatment for patients with diabetes, efforts must concentrate on improving islet graft survival and functionality in highly vascularized, nutrient- and oxygen-rich environments [64]. 

While the intrahepatic percutaneous trans-hepatic portal vein approach to islet transplantation is generally regarded as reliable and safe, several associated risks persist. These include the potential for bleeding and portal venous thrombosis, as well as the risk of accidentally puncturing the gallbladder during the procedure. Additionally, mild increases in levels of liver enzymes like alanine transaminase and aspartate transaminase may occur in up to half of patients post-procedure, typically resolving within one month without intervention. Furthermore, transient discomfort or modest pain at the site of catheter insertion, often accompanied by referred pain at the right shoulder tip due to diaphragmatic irritation, may affect around half of patients. Fortunately, these symptoms can be effectively managed with standard analgesic medications, with resolution usually occurring within 24 to 48 h in most cases [62,65].

### 2.5. Teplizumab

Teplizumab is a humanized monoclonal antibody that delays the onset of type 1 diabetes mellitus. It was approved by the FDA in 2022. Teplizumab is the first treatment to change the course of type 1 diabetes mellitus since the discovery of insulin in 2022. Teplizumab became the first drug to be approved to delay any autoimmune disease before clinical onset. This drug specifically delays the onset of clinical type 1 diabetes in stage 3 in adults and children aged 8 years and above, but the use of intravenous teplizumab on newly diagnosed type 1 diabetes is questionable [66]. Teplizumab is a humanized immunoglobulin G1 monoclonal antibody and it has a high affinity to bind with the c chain of CD3. Teplizumab was first explored for treating acute transplant rejection and psoriatic arthritis [67]. Concurrently, studies in both spontaneous and chemically induced diabetic mouse models demonstrated its potential in reversing or preventing autoimmune diabetes and promoting immune tolerance. Unlike previous immune therapies, continuous administration was unnecessary [67].

Teplizumab leads to decreased insulin use in diabetes patients and the area under the curve (AUC) of the c peptide is much higher. It does not have any effect on Hb1c levels [65]. There is an improvement in total and early insulin secretion rates, which identifies a functional as well as a quantitative improvement in insulin release. In addition to quantitative decreases in C-peptide AUC, studies have identified qualitative abnormalities in beta-cell secretory kinetics, with a loss of early insulin secretion reflecting beta-cell dysfunction before the onset of T1D [68]. In summary, teplizumab treatment changes the biological course of the disease by enhancing beta-cell function reflected by the quantitative and qualitative improvements in insulin secretion. These changes were associated with modulation of the frequency and function of memory CD8+ T cells [69].

The primary adverse events observed are lymphopenia, rash, and headache, with most cases occurring during and shortly after the initial weeks of teplizumab treatment. These effects typically resolved on their own without requiring any intervention, reflecting a safety profile marked by temporary adverse events following one or two courses of teplizumab therapy [67].

The study leading to FDA approval for teplizumab was conducted by the Trial Net network, supported by funding from the National Institutes of Health. It aimed to evaluate the impact of a single 14-day infusion course on the progression from stage 2 to stage 3 of type 1 diabetes. Although the trial was relatively small, with 44 participants in the placebo group and 32 in the treatment group, initial findings reported in 2019 indicated that teplizumab infusion delayed the onset of stage 3 type 1 diabetes by a median duration of 24 months. An updated analysis in 2021 revealed that this median delay had extended to 32.5 months [69].

The risks of immunosuppression, side effects, potential long-term safety issues, variable efficacy, high cost, and the need for regular monitoring must be carefully considered when deciding on its use. 

## 3. Potential and Challenges of Emerging Treatments for Type 1 Diabetes Mellitus (T1DM)

Mesenchymal stem cells can differentiate into insulin-secreting cells, modulate immune responses, and promote beta-cell regeneration. Immune rejection, variability in therapeutic outcomes, limited engraftment, and potential for tumorigenesis are significant hurdles. Consistency and long-term efficacy remain uncertain [25,26,28]. MSC-Exos promote beta-cell regeneration, enhance insulin secretion, and exert immunomodulatory effects, making them promising for T1DM management. Standardized isolation, characterization methods, safety, biological stability, and cost-effectiveness are critical issues that need to be addressed [38,39,40]. CRISPR-Cas9 and other gene-editing technologies offer targeted correction of genetic defects and immune modulation, with potential curative effects. Efficient and targeted gene delivery, long-term gene expression, safety concerns (e.g., insertional mutagenesis), ethical and social concerns, regulatory hurdles, and high costs are significant barriers [57,58,59]. Islet cell transplantation restores insulin production, achieving long-term glycemic control in selected patients with unstable T1DM, while donor availability, immune rejection, the need for lifelong immunosuppression, and procedural risks (e.g., bleeding and portal venous thrombosis) limit widespread use [62,64,65]. Teplizumab delays the onset of clinical T1DM, enhancing beta-cell function and modulating immune responses, while side effects (e.g., lymphopenia and rash), variable efficacy, high costs, and the need for regular monitoring pose significant concerns [67].

## 4. Clinical Implications of Emerging Treatments for Type 1 Diabetes Mellitus (T1DM)

Emerging treatments offer potential for better glycemic control by directly addressing underlying pathophysiology (e.g., beta-cell regeneration and immune modulation). They also aim to reduce the daily management burden, potentially improving quality of life, though new challenges (e.g., side effects and monitoring) may arise. They also have potential for reducing long-term complications by restoring endogenous insulin production and achieving better glycemic control. Public awareness about the benefits and availability of novel T1DM treatments can improve acceptance and adoption.

## 5. Conclusions

Emerging treatment modalities for type 1 diabetes mellitus such as mesenchymal stem cells (MSCs), MSC-derived exosomes (MSC-Exos), gene therapy, islet allotransplantation, pancreatic islet cell transplantation, and teplizumab offer significant potential but also present notable challenges and debates. MSCs and MSC-Exos have shown promise in beta-cell regeneration and immune modulation, though issues of variability, purity, and long-term efficacy remain [70]. Gene therapy offers a potentially curative approach by correcting genetic defects but faces technical hurdles such as efficient gene delivery and safety concerns [58]. Islet allotransplantation and pancreatic islet cell transplantation can restore insulin production, yet they are limited by donor availability, immune rejection, and the need for lifelong immunosuppression [71]. Teplizumab, an anti-CD3 monoclonal antibody, shows potential in delaying T1DM onset, though side effects and patient selection issues need to be addressed. Clinical implications of these therapies include potential improvements in glycemic control and quality of life, but they are also constrained by high costs, technical challenges, and regulatory hurdles. Future research should focus on standardizing protocols, improving delivery systems, addressing safety concerns, and ensuring equitable access to these advanced therapies.

## 6. Recommendations

To address the challenges presented by novel modalities in T1DM effectively, collaboration and concerted action among stakeholders are imperative. Healthcare professionals should prioritize comprehensive education and training to adeptly use emerging treatments and technologies. Collaboration among researchers, clinicians, industry partners, and patient advocacy groups is crucial for accelerating the development and evaluation of new treatments. Policymakers should advocate for streamlined regulatory pathways to ensure timely approval and access. Additionally, involving patients and caregivers in decision-making processes is essential for patient-centered care. Adequate funding and resources are needed to support research and development, with fair pricing and reimbursement policies to enhance affordability and accessibility. Robust surveillance systems are necessary to monitor safety and efficacy post-market, and public awareness about the benefits of novel T1DM treatments should be raised. Embracing digital health solutions can improve treatment delivery and monitoring. Continued clinical studies and technological advancements may refine these treatments, potentially leading to the effective management or eradication of type 1 diabetes on a global scale.

## Data Availability

No new data were created or analyzed in this study. Data sharing is not applicable to this article.
